# Photobiomodulation using low-level laser therapy (LLLT) for patients with chronic traumatic brain injury: a randomized controlled trial study protocol

**DOI:** 10.1186/s13063-017-2414-5

**Published:** 2018-01-08

**Authors:** Guilherme da Cruz Ribeiro Poiani, Ana Luiza Zaninotto, Ana Maria Costa Carneiro, Renato Amaro Zangaro, Afonso Shiguemi Inoue Salgado, Rodolfo Borges Parreira, Almir Ferreira de Andrade, Manoel Jacobsen Teixeira, Wellingson Silva Paiva

**Affiliations:** 1 Division of Psychology at Hospital of Clinics, University of Sao Paulo Medical School, Sao Paulo, Brazil; 2 Division of Neurosurgery, University of Sao Paulo Medical School, Av. Dr. Arnaldo, 455 - Cerqueira César, 01246-903 Sao Paulo, SP Brazil; 3 Institute of Biomedical Engineering, Anhembi Morumbi University, Sao Jose dos Campos, Sao Paulo, Brazil; 4 Salgado Institute of Integral Health; School of Postural and Manual Therapy, Londrina, Parana Brazil; 5 Center for Innovation, Technology and Education – CTE, Sao Jose dos Campos, Sao Paulo, Brazil

**Keywords:** Traumatic brain injury, Cognition, Memory, Depression, Anxiety, Photobiomodulation, Rehabilitation

## Abstract

**Background:**

Photobiomodulation using low-level laser therapy (LLLT) has been tested as a new technique to optimize recovery of patients with traumatic brain injury (TBI). The aim of this study is to evaluate inhibitory attentional control after 18 sessions of active LLLT and compare with the placebo group (sham LLLT). Our exploratory analysis will evaluate the efficacy of the active LLLT on verbal and visuospatial episodic memory, executive functions (working memory, verbal and visuospatial fluency, attentional processes), and anxiety and depressive symptoms compared to the sham group.

**Methods/Design:**

A randomized double-blinded trial will be made in 36 patients with moderate and severe TBI. The active LLLT will use an optical device composed of LEDs emitting 632 nm of radiation at the site with full potency of 830 mW. The cranial region with an area of 400 cm^2^ will be irradiated for 30 min, giving a total dose per session of 3.74 J/cm^2^. The sham LLLT group contains only an LED device with power < 1 mW, only serving to simulate the irradiation. Each patient will be irradiated three times per week for six weeks, totaling 18 sessions. Neuropsychological assessments will be held one week before the beginning of the sessions, after one week, and three months after the end of LLLT sessions. Memory domain, attention, executive functioning, and visual construction will be evaluated, in addition to symptoms of depression, anxiety, and social demographics.

**Discussion:**

LLLT has been demonstrated as a safe and effective technique in significantly improving the memory, attention, and mood performance in healthy and neurologic patients. We expect that our trial can complement previous finds, as an effective low-cost therapy to improve cognitive sequel after TBI.

**Trial registration:**

ClinicalTrials.gov, NCT02393079. Registered on 20 February 2015.

## Background

### Epidemiology

Traumatic brain injury (TBI) is one of the main problems in the public health system due to its magnitude and clinical and social consequences [1]. It is the main cause of disability in young adults, with an estimated 2 million visits to emergency services in the United States in 2009 [2]. In low-income countries, the rate of TBI, especially due to motor vehicles, are > 80% of all cases, leading to higher costs and disabilities [3, 4]. In Brazil, the incidence of TBI is 65.6/100,000 inhabitants per year; however, this incidence appears to be underestimated due to the lack of methodology or inadequate documentation of medical records [5].

### Clinical and functional problems due to TBI

TBI is an alteration in brain function, or other evidence of brain pathology, caused by an external cause [6]. The trauma can result in long-lasting disability, even in people with mild TBI [7].

Long-term neurological and cognitive deficits are expected, including a variety of cognitive impairments, such as in learning, executive function including working memory [8, 9] and verbal fluency [10], reaction time, perceptual organization [11], attention, verbal and visual episodic memory [12, 13], depression, anxiety [8, 9, 14], fatigue, sleep disorders, and attention deficit [15]. Those symptoms are associated with loss of functionality,absence from work, and personal and social privations [16, 17].

### Rationale for the photobiomodulation using low-level laser therapy (LLLT) in patients with TBI

Considerable evidence has shown that the brain has an extensive ability of reorganization after damage [18]. Non-invasive brain stimulation (NIBS) can modulate brain plasticity after trauma through increasing synaptic strength, modulating neurotransmitters, and modifying neural networks [18–21]. LLLT is a technique of NIBS that the irradiation of specific infrared wavelengths is able to penetrate deeply into the brain [22]. These effects produce many biological responses, such as affecting the forming of adenosine triphosphate (ATP), increasing deoxyribonucleic acid (DNA) and ribonucleic acid (RNA), releasing nitric oxide (NO), cytochrome c oxidase (CCO), regulating reactive oxygen species (ROS), and modifying intracellular organelle membrane activity particularly in mitochondria, calcium flux, and stress proteins [23–25]. LLLT produces a shift toward higher oxidation in the overall cell redox potential [26] and briefly increases the level of ROS [27]. This change in the redox state of the mitochondria regulates several transcription factors [28]. These include redox factor-1 (Ref-1), cAMP response element (CREB), activator protein 1 (AP-1), p53, nuclear factor kappa B (NF-jB), hypoxia-inducible factor (HIF-1), and HIF-like factor [28]. The activation and regulation of redox-sensitive genes and transcription factors are thought to be caused by ROS induced from LLLT [27]. In turn, both ATP levels and blood flow increase, improving oxygenation found in damaged areas of the brain [28]. In animal models, LLLT has been investigated as an alternative treatment for brain injury, increasing neurogenesis after TBI [29] and showing a protective effect for stroke [30] and benefits for acute ischemic stroke, acute myocardial infarction, injured peripheral nerves, and spinal cord injury [31–33]. Previous studies showed the safety of photobiostimulation in humans [34], including promising interventions for acute stroke [34], TBI [17, 35–37], and dementia [38]. However, to date, just one open-label clinical trial studies the effect of LED therapy on improving cognitive function in patients with TBI [37]. Likewise, no randomized controlled trial studied the short-term and long-term effects of LLLT on the cognition of closed TBI patients.

### Objective

The aim of this study is to evaluate inhibitory attentional control after 18 sessions of active LLLT and compare with the placebo group (sham LLLT). Our exploratory analysis will evaluate verbal and visuospatial episodic memory, executive functions (working memory, verbal and visuospatial fluency, attentional processes), and anxiety and depressive symptoms.

## Methods

### Study design

**Fig. 1:**
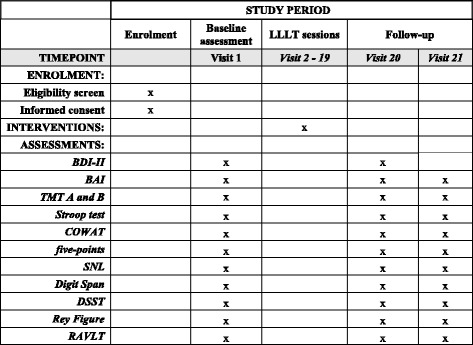
Standard Protocol Items: Recommendations for Interventional Trials (SPIRIT): enrollment, assessment, interventions, and data collection. BDI-II: Beck Depression Inventory, 2^nd^ edition; BAI: Beck Anxiety Inventory; TMT A and B: Test Trail Making form A and B; COWAT: Controlled Oral Word Association Test; SNL: Sequence of Numbers and Letters; DSST: Digit Symbol Substitution Test; RAVLT: Rey Auditory Verbal Learning Test

**Fig. 2:**
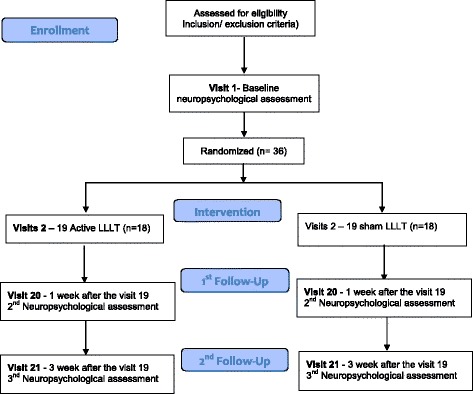
Flowchart of the LLLT study

This is a prospective, multicenter, randomized, parallel placebo-controlled trial that will be conducted at the Clinics Hospital, University of São Paulo (HC-FMUSP) and Salgado Institute, Londrina, Brazil. The protocol is registered on clinicaltrials.org (number NCT02393079). The trial will follow the CONSORT (Consolidated Standards of Reporting Trials) guidelines as well as SPIRIT (Standard Protocol Items: Recommendations for Interventional Trials) guidelines (Fig. 1). Figure 2 provides all information about patient enrollment, interventions, and follow-ups.

### Eligible participants

#### Inclusion criteria

- Patients of both genders;
- Age range 18–60 years;
- Glasgow Coma Scale (GCS) ≤ 12 at admission in the emergency room;
- Computer tomography (CT) or magnetic resonance imaging (MRI) consistent with closed TBI, including intracranial hematoma, subdural hematoma, epidural hematoma, diffuse axonal injury, hemorrhagic contusion, and subarachnoid hemorrhage;
- Loss of consciousness ≥ 30 min;
- Post-traumatic amnesia of ≥ 24 h;
- Outpatients with > 6 months TBI;
- Signed informed consent.

#### Exclusion criteria

- Metal implant or device in the brain or scalp;
- Uncontrolled epilepsy;
- Non-consent sign;
- Portuguese not their first language;
- Non-comprehension and/or not able to follow instructions.

#### Recruitment

The patients will be contacted and invited to participate before or after their regular appointment at the Neurotrauma Outpatient Clinics at the HC-FMUSP or by a contact list of provided by the acute Neurotrauma Inpatient Section at the HC-FMUSP. The patients will give verbal consent before attending their first consent visit.

### Study intervention

#### Standard care

All patients will keep their clinical follow-up at the Neurotrauma Outpatient Clinics at HC-FMUSP or at Salgado Institute, Brazil, independently of the study group assignment or decision of dropping out. A trained research nurse and a psychologist introduced the trial to patients informing them of the principles, objectives, and safety/harm. A trained physical therapist will also conduct and follow the patients along the trial.

All information related to patients (i.e. data analysis, anthropometrical measures, reports, and other relevant information) will be stored in locked file cabinets in areas with limited access. These files will be identified with a code ID and only researchers of this study will have access to this information.

#### LLLT intervention

**Fig. 3:**
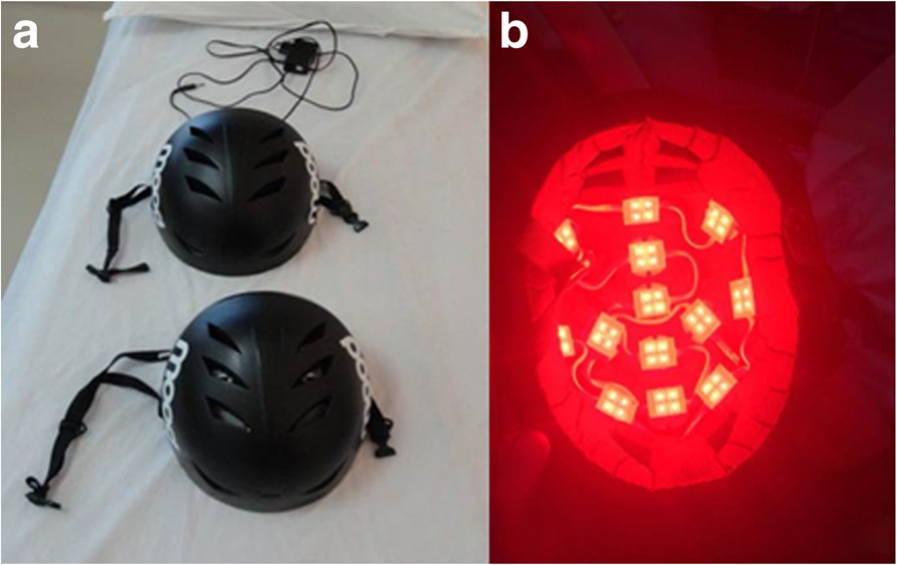
**a** Two identical helmets, active and sham stimulation. **b** The inside view of the active LLLT helmet

Two optical and identical devices (helmets) will be used for the intervention (Fig. 3). For the active optical device, a helmet configured with 13 sets of LEDs at a 632-nm wavelength will distribute a total optical radiation of 830 mW over the skull surface. The skull area receiving irradiation is approximately 400 cm^2^ during 30 min, in which the total expected dose per session is 3.74 J/cm^2^. However, only 3.1% of this energy reaches the gray matter [39].

The rationale follows: optimal wavelength penetration within biological tissues, occurs with a wavelength range of 600–1000 nm [40]. The inactive helmet (sham) contains only LEDs with power < 1 mW, only serving to simulate the irradiation of the patient. All the patients will be submitted to 18 30-min LLLT sessions (active or sham condition), three times per week over six weeks. For safety, after the LLLT intervention, the patient will be asked about side effects between sessions.

#### Neuropsychological assessment

Patients will be assessed at baseline, one week after the end of the LLLT, and three months after the LLLT sessions. Each patient will follow the participation period as shown in Fig. 1.

Instruments:

- Beck Depression Inventory (BDI-II), used to assess depressive symptoms, classified as minimal (0–11 scores), mild (12–19), moderate (20–35), and severe (36–63) [41, 42];
- Beck Anxiety Inventory (BAI), used to assess anxiety symptoms, classified as minimal (0–7 scores), mild (8–15), moderate (16–25), and severe (26–63) [42];
- Trail Making Test A and B (TMT A and TMT B), used to measure attention, speed, and mental flexibility. In TMT A, the patient is instructed to draw a line from 1 to 2, 2 to 3, and so on. In TMT B, the patient switches between numbers and letters, drawing a line from 1 to A, A to 2, 2 to B, and so on. On both forms A and B, the patients perform a sample before the real test. The patient is instructed to perform as fast as he/she can [43];
- Stroop Test - Version Victoria, measuring cognitive control with each person can maintain a goal in mind and suppress a habitual response in favor of a less familiar one. The interference effect is determining by calculating the extra time required to name colors (Card 3) in comparison to the time required to name colors in the control task (Card 1) [43, 44];
- Controlled Oral Word Association Test (COWAT), used to evaluate the spontaneous production of words under restricted search conditions (verbal association fluency). For the phonemic fluency task, the patient must provide orally as many words as possible beginning with the letters F, A, and S for 1 min for each letter. For the semantic fluency task, the patient is asked to produce as many animals’ names as possible within 1 min. The total correct is the sum of all admissible words for the semantic category [43];
- Five-point test, this test requires preproduction of novel designs under time constraints (3 min). The score is calculated including the total number of unique designs and the number of repeated designs (perseverations) [45];
- Symbol Digit Modalities Test (SDMT) oral version, the test is used to assess divided attention, visual scanning, tracking, and speed. The examiner records the numbers that correspond with symbols. The number of correct substitutions within the 90-s interval is the score of the task [46, 47];
- Rey Auditory Verbal Learning Test (RAVLT), measures immediate episodic memory, learning, susceptibility to interference, and recognition memory. A list of 15 nouns (list A) is read aloud to the patient, for five consecutive trials, each followed by a free-recall test. An interference list of 15 words (list B) is presented, followed by a free-recall test of that list. Immediately after this, delayed recall of list A is tested (Trial 6), and after 20 min (Trial 7), without further presentation of those words. The recognition is performed by presenting 50 words containing words from lists A, B, and new words, and the patient needs to identity the words from list A [43, 48];
- Sequence Numbers and Letters (Wechsler subscale - WAIS-III), used to evaluate working memory, random numbers, and letters is presented orally to the patient and must be mentally organized and immediately reported to the examiner with the following rule: first the crescent numbers then the letters (in alphabetical order). In test difficulty increases the patients correctly perform the task [49];
- Digit Span (DS) Forward and Backwards (WAIS-III), the DS assesses working memory. For the DS forward, the patient hears carefully a sequence of numbers and must repeat it immediately in the same sequence as previously presented. For the DS backwards, the patient must repeat the sequence inversely. In both conditions the number of numbers presented increases as the patient hits two corrects scores from the same span [49];
- Rey-Osterrieth Complex Figure Test (ROCF), the purpose is to assess visual–spatial constructional ability and visual memory. The patient is required to copy the ROCF, and after 3 and 30 min, a white sheet is given to the patient, and asked to draw the amount of information retained over time [50, 51].

### Primary outcome

- Inhibitory attentional control (time score) measured by Stroop test. The Stroop test is known to be an accurate assessment of executive function in mild to moderate TBI [52].

### Secondary outcomes

- Verbal and visuospatial episodic memory;
- Executive functions (working memory, verbal and visuospatial fluency, attentional processes);
- Anxiety and depressive symptoms.

### Sample size

Based on the previous pilot study [37] that analyzed the cognitive function of patients with TBI after repetitive sessions of RED/Near-infrared LED treatment. Considering the improvement on Stroop test of 1 standard deviation (SD) related to the baseline, the level of significance of 0.05 and the power of 80%, 16 patients per group was estimated. Considering the rate of 15% of drop-outs, we added two participants per group, ending with a total sample of 36.

### Randomization

All eligible recruited patients will be randomly divided into two groups with a 1:1 ratio and blocks of four and six performed by the co-investigator through the randomization list (randomization.com). The randomization list will be kept in safe storage with the co-investigator responsible for the stimulation sessions. She will provide the allocation concealment assignment for the patients. No information about the assigned group will be given for the patient or to the research assessor.

### Blinding and allocation concealment

Both helmets, the active and the sham, are identical in size, color, and weight. The active helmet activates the red light when plugged; however, the sham helmet cannot provide visible light. The patient is not able to see the light and there is no hit during the active stimulation that can suggest the type of intervention. The unblinded investigator is the person in charge of performing the stimulation. Both assessor researcher and patient will be blind for the type of intervention.

During the study, telephone contact can be used to maintain patient adherence to the trial. This study involves the participation of a research committee not directly related to the allocation of the patients. They can remove the blinding if any relevant situation may rise involving any clinical condition, adverse event, or even abandonment of the patient.

### Safety considerations and adverse events

Phase 1 trials and animal studies showed evidence related to the safety of the LLLT [24, 26, 53]. Although there are few studies in clinical patients, no side effects were reported using photostimulation and LLLT [17, 22, 37, 38, 54–57]. For safety, the LLLT will be applied by a registered nurse or a trained clinician.

### Ethical approval and consent to participate

The protocol was approved by CONEP Resolution 466/2012, which regulates scientific research involving human beings in Brazil. It follows the ethical principles of Declaration of Helsinki for medical research involving human subjects.

### Statistical analysis

Intention-to-treat and per-protocol analyses will be performed for the primary and secondary outcomes. Missing data will be analyzed with regression imputation, considering the confounders (months of the trauma, years of age, schooling years, and depressive symptoms). Patients that did not receive 60% of the total stimulation will be considered non-adherent and analyzed as per-protocol.

Means and standard deviation will be used to represent data with normal distribution and medians and quartiles to describe non-normally distributed data. Two-way ANOVA (2 groups × 3 times) with repeated measures (for parametric variables) or the Kruskal–Wallis test (for non-parametric variables) will be used for the analysis of the TLT effects obtained in the three timelines: baseline, one week, and three weeks after the intervention. The Bonferroni correction for multiple comparisons will be employed as a post-hoc test. The effect size will be calculated based on the difference between means of the pre-intervention and post-intervention evaluations and will be expressed with respective 95% confidence intervals. Parametric Student’s t-test or a Mann–Whitney *U* test for non-parametric data will be employed to assess between-group (active and sham helmet) differences in age, height, weight, and body mass index (BMI), time of TBI injury, level of education, and Glasgow Coma Scale at admission on the emergency room. For all effects, a *p* value < 0.05 will be considered indicative of statistical significance. The data will be organized and tabulated using the Statistical Package for the Social Sciences (SPSSv.19.0).

## Discussion

The present study is designed to evaluate the effects of 18 sessions of LLLT over cognition, in patients with chronic moderate and severe TBI. Our primary hypothesis is that the sessions of active LLLT will improve attention (at least 1 SD) measured by Stroop test compared to the placebo group (sham LLLT). Our secondary hypothesis is that the active group will improve in all domains assessed by the neuropsychological battery, including cognition and mood, compared to placebo group.

LLLT can modulate many biological effects penetrating the scalp into the brain [25], playing a role improving the outcome of the patients in two different ways, depending on the stage of the trauma. In the acute phase after TBI, the initial neuronal injury occurs instantly and oftentimes causes irreversible damage to the central nervous system, due to impairment of neuronal cell functions, including mitochondria, and glia cells [18, 58]. The disruption of neuronal circuitry causes loss of connectivity between different areas of the brain and can negatively impact neural regeneration, leading to dysfunctional interactions. In the secondary stage after the trauma, other changes may occur, including release of neurotransmitters, decreased glucose utilization, lactic acid accumulation, reduced activity of ATP-reliant ion pumps, increased release of glutamate, Ca2 + −induced depolarization, and excitotoxicity [21, 58].

Previous studies showed improvement on cognition including attentional process and episodic memory after repetitive sessions of LLLT in patients with TBI [17, 37]. It seems that the LLLT decreases the inflammatory response, helping the neuroprotection after TBI. This process leads to increases on the ATP, cellular energy, and blood flow, decreasing the metabolic process. In summary, the LLLT seems to increase the intercellular synapses, acting as a possible treatment after acute and chronic TBI [37, 59–61].

Other studies reported the effects of the LLLT in another sample. Lampl et al. [34] used the LLLT in patients with ischemic stroke and showed that infrared wavelength therapy was safe and effective in the experimental group compared to the control groups when treatment was started 24 h after the onset of stroke. Another study with psychiatric patients showed that seven (out of ten) patients with severe cases of depression and anxiety presented symptom remission after two weeks elapsed with four LLLT applications in the prefrontal region, which did not occur with the control group [62]. Likewise, LLLT has been demonstrated as a safe and effective technique in significantly improving the memory, attention, and mood performance of healthy people [63]. Overall, we expect that our trial can complement previous findings, as an effective low-cost therapy to improve cognitive sequel in patients with chronic TBI.

## Trial status

The randomization of patients was started on January 2015. The inclusion of participants is ongoing, with 12 patients completing the assessment, four patients in the second assessment and two patients in the first assessment. We expect to conclude the study by 5 October 2018.

## Abbreviations

AP-1: Activator protein 1
ATP: Adenosine triphosphate
BAI: Beck Anxiety Inventory
BDI-II: Beck Depression Inventory II
BMI: Body mass index
CCO: Cytochrome c oxidase
CONEP: National Commission for Research Ethics
CONSORT: Consolidated Standards of Reporting Trials
COWAT: Controlled Oral Word Association Test
CREB: cAMP Response Element-Binding Protein
CT: Computed tomography
DNA: Deoxyribonucleic acid
DS: Digit span
DSST: Digit Symbol Substitution Test
GCS: Glasgow Coma Scale
HIF-1: Hypoxia inducible factor
LED: Light emitting diode
LLLT: Low-level laser therapy
MRI: Magnetic resonance imaging
NF-j B: Nuclear factor Kappa B
NIBS: Non-invasive brain stimulation
NO: Nitric oxide
RAVLT: Rey Auditory Verbal Learning Test
RCT: Randomized controlled trial
Ref-1: Redox factor-1
RNA: Ribonucleic acid
ROCF: Rey-Osterrieth Complex Figure Test
ROS: Reactive oxygen species
SDMT: Symbol Digit Modalities Test
SPIRIT: Standard Protocol Items: Recommendations for Interventional Trials
TBI: Traumatic brain injury
TMT A and B: Test Trail Making form A and B
WAIS III: Wechsler Adult Intelligence Scale III

## References

[1] Kushner DS, Johnson-Greene D (2014). Changes in cognition and continence as predictors of rehabilitation outcomes in individuals with severe traumatic brain injury. J Rehabil Res Dev..

[2] Coronado VG, McGuire LC, Sarmiento K, Bell J, Lionbarger MR, Jones CD (2012). Trends in traumatic brain injury in the U.S. and the public health response: 1995–2009. J Safety Res.

[3] Peeters S, Blaine C, Vycheth I, Nang S, Vuthy D, Park KB (2017). Epidemiology of traumatic brain injuries at a major government hospital in Cambodia. World Neurosurg..

[4] Kamal VK, Agrawal D, Pandey RM (2016). Epidemiology, clinical characteristics and outcomes of traumatic brain injury: Evidences from integrated level 1 trauma center in India. J Neurosci Rural Pract..

[5] de Almeida CER, de Sousa JL, Dourado JC, Gontijo PA, Dellaretti MA, Costa BS (2016). Traumatic brain injury epidemiology in Brazil. World Neurosurg..

[6] Menon DK, Schwab K, Wright DW, Maas AI (2010). Demographics and Clinical Assessment Working Group of the International and Interagency Initiative Toward Common Data Elements for Research on Traumatic Brain Injury and Psychological Health. Position statement: definition of traumatic brain injury. Arch Phys Med Rehabil.

[7] Corrigan JD, Selassie AW, Orman JA (2010). The epidemiology of traumatic brain injury. J Head Trauma Rehabil..

[8] Niogi SN, Mukherjee P, Ghajar J, Johnson CE, Kolster R, Lee H (2008). Structural dissociation of attentional control and memory in adults with and without mild traumatic brain injury. Brain..

[9] Skandsen T, Finnanger TG, Andersson S, Lydersen S, Brunner JF, Vik A (2010). Cognitive impairment 3 months after moderate and severe traumatic brain injury: a prospective follow-up study. Arch Phys Med Rehabil..

[10] Zaninotto AL, Guirado VMD, Baldivia B, Nunes MD, Amorim RL, Teixeira MJ (2014). Improvement of verbal fluency in patients with diffuse brain injury over time. Neuropsychiatr Dis Treat..

[11] Costa TL, Zaninotto ALC, Benute GG, De Lucia MC, Paiva WS, Wagemans J (2015). Perceptual organization deficits in traumatic brain injury patients. Neuropsychologia..

[12] Zaninotto AL, Vicentini JE, Solla DJF, Silva TT, Guirado VM, Feltrin F (2017). Visuospatial memory improvement in patients with diffuse axonal injury (DAI): a 1-year follow-up study. Acta Neuropsychiatrica..

[13] Kinnunen KM, Greenwood R, Powell JH, Leech R, Hawkins PC, Bonnelle V (2011). White matter damage and cognitive impairment after traumatic brain injury. Brain..

[14] Zaninotto AL, Vicentini JE, Fregni F, Rodrigues PA, Botelho C, de Lucia MC (2016). Updates and current perspectives of psychiatric assessments after traumatic brain injury: a systematic review. Front Psych..

[15] Sinclair KL, Ponsford JL, Taffe J, Lockley SW, Rajaratnam SM (2014). Randomized controlled trial of light therapy for fatigue following traumatic brain injury. Neurorehabil Neural Repair..

[16] Esbjornsson E, Skoglund T, Mitsis MK, Hofgren C, Larsson J, Sunnerhagen KS (2013). Cognitive impact of traumatic axonal injury (TAI) and return to work. Brain Inj..

[17] Naeser MA, Saltmarche A, Krengel MH, Hamblin MR, Knight JA (2011). Improved cognitive function after transcranial, light-emitting diode treatments in chronic, traumatic brain injury: two case reports. Photomed Laser Surg..

[18] Li SS, Zaninotto AL, Neville IS, Paiva WS, Nunn D, Fregni F (2015). Clinical utility of brain stimulation modalities following traumatic brain injury: current evidence. Neuropsychiatr Dis Treat..

[19] Fitzgerald PB, Hoy KE, Maller JJ, Herring S, Segrave R, McQueen S (2011). Transcranial magnetic stimulation for depression after a traumatic brain injury: a case study. J ECT..

[20] Lesniak M, Polanowska K, Seniow J, Czlonkowska A (2014). Effects of repeated anodal tDCS coupled with cognitive training for patients with severe traumatic brain injury: a pilot randomized controlled trial. J Head Trauma Rehabil..

[21] Villamar MF, Portilla AS, Fregni F, Zafonte R (2012). Noninvasive brain stimulation to modulate neuroplasticity in traumatic brain injury. Neuromodulation..

[22] Zhang Q, Ma HY, Nioka S, Chance B (2000). Study of near infrared technology for intracranial hematoma detection. J Biomed Opt..

[23] Antunes F, Boveris A, Cadenas E (2004). On the mechanism and biology of cytochrome oxidase inhibition by nitric oxide. Proc Natl Acad Sci U S A..

[24] Khuman J, Zhang J, Park J, Carroll JD, Donahue C, Whalen MJ (2012). Low-level laser light therapy improves cognitive deficits and inhibits microglial activation after controlled cortical impact in mice. J Neurotrauma..

[25] Huang YY, Gupta A, Vecchio D, de Arce VJ, Huang SF, Xuan W (2012). Transcranial low level laser (light) therapy for traumatic brain injury. J Biophotonics..

[26] Karu TI, Pyatibrat LV, Kolyakov SF, Afanasyeva NI (2005). Absorption measurements of a cell monolayer relevant to phototherapy: Reduction of cytochrome c oxidase under near IR radiation. J Photochem Photobiol B Biol..

[27] Chen ACH, Arany PR, Huang YY, Tomkinson EM, Sharma SK, Kharkwal GB (2011). Low-level laser therapy activates NF-kB via generation of reactive oxygen species in mouse embryonic fibroblasts. PloS One..

[28] Thunshelle C, Hamblin MR (2016). Transcranial low-level laser (light) therapy for brain injury. Photomed Laser Surg..

[29] Richardson RM, Sun D, Bullock MR (2007). Neurogenesis after traumatic brain injury. Neurosurg Clin N Am..

[30] Oron U, Yaakobi T, Oron A, Mordechovitz D, Shofti R, Hayam G (2001). Low-energy laser irradiation reduces formation of scar tissue after myocardial infarction in rats and dogs. Circulation..

[31] Leung MCP, Lo SCL, Siu FKW, So KF (2002). Treatment of experimentally induced transient cerebral ischemia with low energy laser inhibits nitric oxide synthase activity and up-regulates the expression of transforming growth factor-beta 1. Lasers Surg Med..

[32] Nissan M, Rochkind S, Razon N, Bartal A (1986). HeNe laser irradiation delivered transcutaneously - its effects on the sciatic nerve of rats. Lasers Surg Med..

[33] Byrnes KR, Waynant RW, Ilev IK, Wu X, Barna L, Smith K (2005). Light promotes regeneration and functional recovery and alters the immune response after spinal cord injury. Lasers Surg Med..

[34] Lampl Y, Zivin JA, Fisher M, Lew R, Welin L, Dahlof B (2007). Infrared laser therapy for ischemic stroke: A new treatment strategy results of the NeuroThera effectiveness and Safety Trial-1 (NEST-1). Stroke..

[35] Naeser MA, Hamblin MR (2015). Traumatic brain injury: a major medical problem that could be treated using transcranial, red/near-infrared LED photobiomodulation. Photomed Laser Surg..

[36] Naeser MA, Martin PI, Ho MD, Krengel MH, Bogdanova Y, Knight JA (2016). Transcranial, red/near-infrared light-emitting diode therapy to improve cognition in chronic traumatic brain injury. Photomed Laser Surg..

[37] Naeser MA, Zafonte R, Krengel MH, Martin PI, Frazier J, Hamblin MR (2014). Significant improvements in cognitive performance post-transcranial, red/near-infrared light-emitting diode treatments in chronic, mild traumatic brain injury: open-protocol study. J Neurotrauma..

[38] Saltmarche AE, Naeser MA, Ho KF, Hamblin MR, Lim L (2017). Significant improvement in cognition in mild to moderately severe dementia cases treated with transcranial plus intranasal photobiomodulation: case series report. Photomed Laser Surg..

[39] Haeussinger FB, Heinzel S, Hahn T, Schecklmann M, Ehlis AC, Fallgatter AJ (2011). Simulation of near-infrared light absorption considering individual head and prefrontal cortex anatomy: implications for optical neuroimaging. PloS One..

[40] Karu TI (1986). Molecular mechanism of the therapeutic effect of low-intensity laser irradiation. Dokl Akad Nauk SSSR..

[41] Beck AT, Ward CH, Mendelson M, Mock J, Erbaugh J (1961). An inventory for measuring depression. Arch Gen Psychiatry..

[42] Beck AT, Steer RA (1993). Beck Anxiety Inventory manual.

[43] Spreen O, Strauss E (1998). A compendium of neuropsychological tests administration norms and commentary.

[44] Stuss DT, Floden D, Alexander MP, Levine B, Katz D (2001). Stroop performance in focal lesion patients: dissociation of processes and frontal lobe lesion location. Neuropsychologia..

[45] Regard M, Strauss E, Knapp P (1982). Children’s production on verbal and non-verbal fluency tasks. Percept Mot Skills..

[46] Burik TE (1950). Relative roles of the learning and motor factors involved in the digit symbol test. J Psychol..

[47] McLeod DR, Griffiths RR, Bigelow GE, Yingling J (1982). An automated version of the digit symbol substitution test (DSST). Behav Res Methods Instrum..

[48] Malloy-Diniz LF, Lasmar VA, Gazinelli LdeS, Fuentes D, Salgado JV. The Rey Auditory-Verbal Learning Test: applicability for the Brazilian elderly population. Revista Brasileira De Psiquiatria. 2007;29:324–9 10.1590/s1516-44462006005000053 17713697

[49] Nascimento E, Figueiredo VL (2005). Escala de Inteligência Weschler para Adultos - manual técnico.

[50] Rey A. Figuras complexas de Rey - manual técnico. São Paulo: Casa do Psicologo; 2010

[51] Tombaugh TN, Faulkner P, Hubley AM (1992). Effects of age on the Rey-Osterrieth and taylor complex figures - test-retest data using an intentional learning-paradigm. J Clin Exp Neuropsychol..

[52] Lezak MD, Howieson DB, Bigler ED, Tranel D (2012). Neuropsychological assessment.

[53] Smith KC (2005). Laser (and LED) therapy is phototherapy. Photomed Laser Surg..

[54] Lavery LA, Murdoch DP, Williams J, Lavery DC (2008). Does anodyne light therapy improve peripheral neuropathy in diabetes? A double-blind, sham-controlled, randomized trial to evaluate monochromatic infrared photoenergy. Diabetes Care..

[55] Rojas JC, Bruchey AK, Gonzalez-Lima F (2012). Low-level light therapy improves cortical metabolic capacity and memory retention. J Alzheimers Dis..

[56] Rojas JC, Gonzalez-Lima F (2013). Neurological and psychological applications of transcranial lasers and LEDs. Biochem Pharmacol..

[57] Naeser M, Ho M, Martin P, Treglia EM, Krengel MH, Hamblin M (2012). Improved language after scalp application of red/near-infrared light-emitting diodes: pilot study supporting a new, noninvasive treatment for chronic aphasia. Procedia Social and Behavioral Sciences..

[58] de Andrade AF, Paiva WS, de Amorim RLO, Figueredo EG, Rusafa Neto E, Teixeira MJ (2009). The pathophysiological mechanisms following traumatic brain injury. Rev Assoc Med Bras..

[59] Naeser MA, Hamblin MR (2011). Potential for transcranial laser or LED therapy to treat stroke, traumatic brain injury, and neurodegenerative disease. Photomed Laser Surg..

[60] Hennessy M, Hamblin MR (2017). Photobiomodulation and the brain: a new paradigm. J Opt..

[61] Lapchak PA (2010). Taking a light approach to treating acute ischemic stroke patients: Transcranial near-infrared laser therapy translational science. Ann Med..

[62] Schiffer F, Johnston AL, Ravichandran C, Polcari A, Teicher MH, Webb RH (2009). Psychological benefits 2 and 4 weeks after a single treatment with near infrared light to the forehead: a pilot study of 10 patients with major depression and anxiety. Behav Brain Funct..

[63] Barrett DW, Gonzalez-Lima F (2013). Transcranial infrared laser stimulation produces beneficial cognitive and emotional effects in humans. Neuroscience..

